# Telomere length and oxidative stress in small cell lung cancer patients: changes through chemotherapy cycles compared to healthy controls

**DOI:** 10.11613/BM.2025.020705

**Published:** 2025-04-15

**Authors:** Azra Guzonjić, Dragana Jovanović, Ivana Simić, Vesna Ćeriman Krstić, Natalija Samardzić, Barbara Ostanek, Janja Marc, Miron Sopić, Jelena Kotur Stevuljević

**Affiliations:** 1 Department for Medical Biochemistry, Faculty of Pharmacy, University of Belgrade, Belgrade, Serbia; 2 Internal Medicine Clinic “Akta Medica”, Belgrade, Serbia; 3 Merck Sharp & Dohme d.o.o., Medical Affairs, Belgrade, Serbia; 4 Faculty of Medicine, University of Belgrade, Belgrade, Serbia; 5 Clinic for Pulmonology, University Clinical Center of Serbia, Belgrade, Serbia; 6 Department of Clinical Biochemistry, Faculty of Pharmacy, University of Ljubljana, Ljubljana, Slovenia

**Keywords:** telomere length, redox status, small cell lung cancer, cisplatin/etoposide regimen

## Abstract

**Introduction:**

Small cell lung cancer (SCLC) is an aggressive malignant disease with poor survival outcomes. The aim of this study was to investigate leukocyte telomere length (LTL) and redox status parameters during chemotherapy and evaluate their prognostic potential based on the hypothesis that shorter LTL and oxidative stress burden correlate with poorer survival.

**Materials and methods:**

This longitudinal study included 60 SCLC patients and 73 healthy controls. Leukocyte telomere length was measured by quantitative PCR (qPCR) method, while redox status parameters (MDA - malondialdehyde, IMA - ischemia-modified albumin, PON1 - paraoxonase 1, redox index) were determined by spectrophotometric methods before, after two and after four cycles of chemotherapy.

**Results:**

All measured parameters showed significant differences between patients and controls, except the oxy-score (P < 0.001). Significant differences in IMA, PON1 and redox index were observed between SCLC patient groups at different time points (P < 0.001). Significant differences in IMA and PON1 were observed between SCLC survival groups, with higher values found in survivors after two chemotherapy cycles (P < 0.001). Redox index was the highest in the pre-chemo group (P = 0.019). Among patients who died, PON1 activity differed significantly between those who died within 2 months and after 4 months (P = 0.028). Kaplan-Meier analysis showed that LTL and PON1 were significant predictors of survival, with values below the 25th percentile associated with a higher risk of death.

**Conclusions:**

Leukocyte telomere length and PON1 are potential prognostic biomarkers for SCLC survival, suggesting their potential use in non-invasive biomarker panels for improved patient stratification.

## Graphical abstract

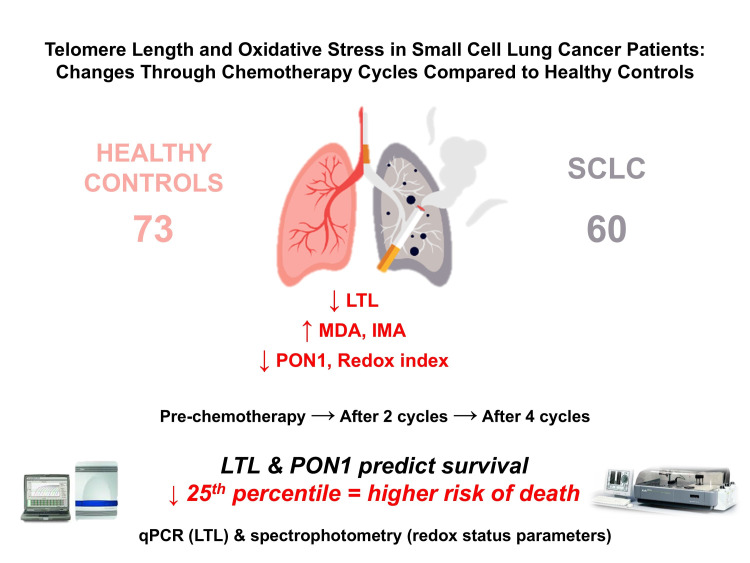

## Highlights

• Shorter telomeres are observed in small cell lung cancer group

• Malondialdehyde and ischemia*-*modified albumin are higher in small cell lung cancer

• Paraoxonase 1 activity is reduced in small cell lung cancer

• Telomere length and paraoxonase 1 activity predict survival in small cell lung cancer

## Introduction

Small cell lung cancer (SCLC) is an aggressive malignancy, accounting for 15% of lung cancers, with rapid growth, early metastasis, and poor prognosis (*1*). Lung cancer remains the leading cause of cancer-related deaths, responsible for 1.8 million deaths annually (*2*). The five-year survival rate is 2-5% in the extensive stage and 15-30% in the limited stage (*1*). Smoking is the primary risk factor, along with occupational exposure, air pollution, and genetic predisposition (*3*). First-line treatment involves platinum-based chemotherapy with etoposide, but most patients relapse and develop resistance (*1*). Identifying biomarkers for treatment response and disease progression is crucial, though the roles of telomere length and oxidative stress in SCLC remain unclear.

Telomeres, which protect chromosome ends from damage and degradation, shorten over time, leading to cell senescence and signaling necrosis or apoptosis (*4*). In a cancer cell, death does not occur despite critically short telomeres, but the cells continue to proliferate, either through increased activity of the telomerase, which lengthens the telomeres, or through another mechanism that is independent of the telomere/telomerase system. Excessive oxidative stress can accelerate the process of telomere shortening. The generated reactive oxygen species (ROS) lead to an oxidative modification of the telomere segment, which is no longer susceptible to elongation by telomerase (*5*). Short telomeres measured in peripheral white blood cells are known to be a risk factor for the development of lung cancer (*6*). Progressive telomere shortening activates the DNA damage response (DDR), triggering signaling pathways and apoptosis in normal cells; however, if these pathways are bypassed, tumorigenesis is allowed to occur, leading to telomere dysfunction (*7*).

The cisplatin/etoposide (PE) regimen exerts its antitumor effect by inducing DNA damage and oxidative stress, leading to apoptosis of cancer cells. Cisplatin generates ROS in a dose- and time-dependent manner, contributing to telomere attrition and genomic instability (*8*). It disrupts G-quadruplex structures, impairs telomere maintenance and accelerates cellular senescence (*9*). Etoposide increases oxidative stress by inhibiting topoisomerase II, which leads to DNA strand breaks and increases ROS concentrations, further promoting telomere dysfunction (*10*). Increased oxidative stress in cancer cells impairs redox homeostasis, a mechanism associated with poor prognosis in SCLC. Studies suggest that oxidative imbalance and telomere shortening play a critical role in treatment response and survival (*11*, *12*).

The main research objective of this study was to determine telomere length in peripheral blood leukocytes and redox status parameters (malondialdehyde - MDA, ischemia-modified albumin - IMA, paraoxonase 1 activity - PON1, and redox index) in serum of SCLC patients (before chemotherapy, after 2 and 4 cycles of chemotherapy) and healthy volunteers. In addition, these parameters were analyzed in relation to the survival status of the patients and clinical indicators of the disease. One of the study objectives was also to assess the predictive or prognostic potential of the measured biomarkers. Better stratification of SCLC patients based on prognostic biomarkers are the focus of our research efforts.

The hypothesis of our research is that leukocyte telomere length (LTL) is a predictor for survival of SCLC patients undergoing platinum-based chemotherapy. We hypothesize that shorter LTL, along with increased oxidative stress and altered redox status parameters, correlate with poorer survival outcomes, suggesting their potential use in non-invasive biomarker panels for improved patient stratification and prediction of chemotherapy response in SCLC.

## Materials and methods

### Subjects

This longitudinal study involved 60 SCLC patients recruited from the University Hospital of Pulmonology, Clinical Center of Serbia, Belgrade, in the period between October 2020 and February 2022. The study was ethically approved by the Ethics Committee of the Faculty of Medicine University of Belgrade under Decision No. 1322/II-81 and by the Ethics Committee of the Faculty of Pharmacy University of Belgrade under Decision No. 2760/3. All subjects were informed about the main objectives and protocol of the study and signed a written informed consent. Inclusion criteria for SCLC group were pathophysiologically confirmed III/IV stage SCLC with an indication for platinum-based chemotherapy. All patients received a cisplatin/etoposide (PE) regimen and were followed at baseline (pre-chemo group), after 2 cycles of chemotherapy (chemo-2 group), and after 4 cycles of chemotherapy (chemo-4 group). In addition, 73 healthy subjects were enrolled in the study as a control group (CG). The inclusion criteria for CG were that they were free of chronic diseases, cancer and recent infections and were not receiving immunosuppressive treatment. We also ensured that the CG had no history of malignancies and no significant health conditions that could influence the results of the study. This study builds on our previous work published in Biomedicines, in which we investigated the correlations between neutrophil-to-lymphocyte ratio (NLR), C-reactive protein (CRP), soluble programmed cell death ligand 1 (sPD-L1) and Schlafen 11 (SLFN11) with response to first-line chemotherapy in a cohort of SCLC patients (*13*). In this follow-up study, we aim to further elucidate the dynamics of telomere length and oxidative stress in the same cohort of patients to improve our understanding of their role in the pathophysiology of SCLC.

### Methods

Peripheral venous blood was collected after overnight fasting in 6 mL BD Vacutainer EDTA Tubes (Becton, Dickinson and Company, Franklin Lakes, USA) and 6 mL BD Vacutainer SST Tubes (Becton, Dickinson and Company, Franklin Lakes, USA) at the University Hospital of Pulmonology, Clinical Center of Serbia, Belgrade, and transported at + 4 °C to the Department of Medical Biochemistry, Faculty of Pharmacy, University of Belgrade, where all analyses were performed. Serum and plasma were collected after centrifugation at 3000 rpm for 10 minutes (Univerzal Z 300, Hermle Labortechnik GmbH, Wehingen, Germany). In addition, peripheral blood mononuclear cells (PBMCs) were isolated from the buffy coat with Ficoll-Paque PLUS (GE Healthcare, Waukesha, USA) and stored at -80 °C until DNA isolation. Genomic DNA was isolated using the FlexiGene DNA Kit (Qiagen, Hilden, Germany). Samples were collected at three different time points: before the initiation of chemotherapy, after 2 cycles of chemotherapy, and after 4 cycles of chemotherapy.

Leukocyte telomere length was measured from isolated genomic DNA by the modified Cawthon real-time quantitative polymerase chain reaction (RTq-PCR) technique using Applied Biosystems 7500 Real-Time PCR System (Thermo Fisher Scientific, Waltham, USA) (*14*). Fluorescent signal proportional to the mean telomere length (T) in the sample was quantified, and compared to the ’’single copy gene’’ (S). Leukocyte telomere length was calculated as a T/S ratio while albumin was used as a normalization gene. The pool was made from isolated DNA samples and used for the preparation of standard dilutions (50, 25, 12.5, 6.25, 3.125, 1.5625, and 0.78125 ng/µL). Each standard and sample was run in triplicate. The protocol for the preparation of the master mix is shown in Supplementary Table 1. The cycling protocol for telomeres and albumin includes an initial activation at 95 °C for 12 minutes, followed by 4 cycles of denaturation at 95 °C for 15 seconds and an annealing step at 49 °C for 20 seconds (for telomeres only). The main cycle includes 40 cycles of denaturation at 95 °C for 15 seconds and annealing at 60 °C for 10 seconds, with elongation at 72 °C for 35 seconds for telomeres and 87 °C for 35 seconds for albumin.

Redox status parameters were measured from serum using spectrophotometric methods on the Ilab 300+ analyzer (Instrumentation Laboratory, Milan, Italy) or the SPECTROstar nano absorbance microplate reader (BMG LABTECH, Ortenberg, Germany). The methods used for these measurements are in-house methods developed and validated in our laboratory to ensure accuracy, reproducibility and sensitivity in accordance with established protocols. Detailed method specifications can be found in the publications listed below. Malondialdehyde and IMA were measured as parameters of oxidative stress, while PON1 activity and redox index (ratio between total antioxidant status and total oxidative status) were determined as parameters of antioxidant protection. In addition, the Oxy score was calculated to better assess oxidative stress. Malondialdehyde was determined as a thiobarbituric acid reactive substance (TBARS) based on the absorption maximum of the complex of MDA and other TBARS with thiobarbituric acid at 535 nm, while IMA was determined with cobalt chloride and dithiothreitol (*15*, *16*). The intra-assay coefficient of variation (CV) for MDA was 4.8% and the inter-assay CV was 7.2%, indicating consistent performance across analyzes. For IMA, the coefficients of variation were between 2.5% and 4.6%, indicating a high precision of the measurements. The activity of PON1 on the substrate paraoxon was measured kinetically, with an intra-assay CV of 5.4% and an inter-assay CV of 7.7%, which underlines the reliability of this method (*17*). Total antioxidant status (TAS) was determined with ABTS as a chromogen (*18*). Total oxidant status (TOS), which mainly represents serum H_2_O_2_ and lipid hydroperoxides, oxidizes the iron(II)-o-dianiside complex to iron(III) ions, which then form a colored complex with xylenol orange in an acidic environment (*19*). The Oxy score was calculated by subtracting the protective score (average z-score of TAS and sulfhydryl group content) from the damage score (average z-score of TOS and prooxidative-antioxidative balance) (*20*).

The basic lipid profile parameters were determined using routine clinical methods. The analysis was performed using the Mindray BS200E analyzer (Mindray Bio-Medical Electronics Co., Ltd., Shenzhen, China), with serum triglycerides (TC), and high density lipoprotein cholesterol (HDL-C) measured by enzymatic methods, while low density lipoprotein cholesterol (LDL-C) was calculated using the Friedewald equation.

### Statistical analysis

The normality of the distribution of continuous data was tested using the Kolmogorov-Smirnov test for groups with more than 50 patients/data and the Shapiro-Wilk test for groups with less than 50 patients/data. Normally distributed data were presented as mean ± standard deviation, while asymmetrically distributed data were presented as median and interquartile range (25th-75th percentile). Comparisons between SCLC groups were tested using a non-parametric ANOVA for repeated measures (Friedman test) and a *post-hoc* Wilcoxon signed-rank test. Comparisons between SCLC groups and CG were performed with a Kruskal-Wallis non-parametric ANOVA and a *post-hoc* Mann-Whitney U-test. Categorical data were presented as absolute and relative frequencies and compared using the Chi-square test. Correlations between selected parameters were tested using Spearman’s bivariate correlation analysis. Principal component analysis (PCA) and univariate binary logistic regression were used to examine the predictive power of the studied variables. Kaplan-Meier survival analysis was used to estimate the survival function of the risk values of the selected parameters. Statistical analyses were performed using IBM SPSS Statistics 29, Premium Edition (IBM Corporation, Armonk, USA), under an institutional license. The P-value < 0.05 was considered statistically significant.

## Results

The sociodemographic, anthropometric, clinical, and smoking-related data of the SCLC cohort and the healthy control group are shown in Table 1. The study included 60 patients (median age 66 years, with 37 males and 23 females), most of whom were current or former smokers with a median smoking duration of 40 years. Tumor characteristics were predominantly T4 tumors and N2 lymph node involvement, with more than half of the patients having limited stage disease. The Eastern Cooperative Oncology Group (ECOG) performance status was predominantly 1. Response to treatment after two cycles showed partial response in 25 out of 60 patients, while disease progression was seen in 21 patients. The survival rate after six months was observed in 25 out of 60 patients (Table 1).

**Table 1:** Clinical and demographic profile of SCLC patients and healthy controls

**Variable**	**Category**	**SCLC** **N = 60**	**CG** **N = 73**	**P**
Age (years)	/	66 (61-70)	51 (46-59)	**< 0.001**
Gender	male female	37/60 23/60	9/73 64/73	**< 0.001**
BMI (kg/m^2^)	/	23.5 (21.6-26.5)	26.0 (23.9-29.1)	**0.005**
Smoking status	non-smoker current smoker former-smoker	1/60 43/60 16/60	47/73 22/73 1/73	**< 0.001**
Number of cigarettes per day	/	20 (20-40)	15 (7-20)	**< 0.001**
Duration of smoking (years)	/	40 (30-45)	20 (20-30)	**< 0.001**
Primary tumor size (T)	T0 T1 T2 T3 T4	4/60 3/60 10/60 12/60 31/60	N/A	/
Regional lymph nodes (N)	N0 N1 N2 N3	9/60 6/60 31/60 14/60	N/A	/
Distant metastasis (M)	M0 M1a M1b M1c	32/60 8/60 6/60 14/60	N/A	/
Stage	IIIA IIIB IIIC IVA IVB	13/60 16/60 3/60 14/60 14/60	N/A	/
Limited stage/Extensive stage	LS ES	32/60 28/60	N/A	/
ECOG performance status	0 1 2	1/60 55/60 4/60	N/A	/
Response after 2 months	complete response partial response stable disease progressive disease	2/60 25/60 12/60 21/60	N/A	/
Response after 4 months	complete response partial response stable disease progressive disease	2/39 13/39 11/39 13/39	N/A	/
Survival status	survivor deceased	25/60 35/60	N/A	/
Time in study	2 months 4 months 6 months	10/60 25/60 25/60	N/A	/
Categorical data are presented as proportion (N/total), while numerical data are presented as median (interquartile range). Data were compared using the Chi-square test of independence for categorical variables and the Mann-Whitney U test for numerical variables. P < 0.05 was considered statistically significant. SCLC - small cell lung cancer. CG - control group. N/A - not applicable. BMI - body mass index. LS - limited stage. ES - extensive stage. ECOG Performance Status - Eastern Cooperative Oncology Group Performance Status				

The comparison of LTL and the redox status parameters studied between the patient groups and the healthy subjects is presented in Table 2, while Table 3 presents the results of a comparative analysis of the aforementioned parameters between the patient subgroups formed on the basis of their survival status.

**Table 2:** Comparison of telomere length and redox status parameters in SCLC patients at different chemotherapy time points and healthy controls

**Parameter**	**CG** **N = 73**	**Pre-chemo** **N = 60**	**Chemo-2** **N = 36**	**Chemo-4** **N = 25**	**P**	P*
LTL	1.03 (0.79-1.28)	0.78 (0.56-0.99)^§^	0.85 (0.63-1.07)^†^	0.67 (0.53-0.83)^§. ††^	**< 0.001**	0.170
MDA (μmol/L)	2.590 (2.220-3.040)	3.926 (2.963-4.519)^§^	3.407 (2.963-4.555)^§^	3.185 (2.667-6.037)^§^	**< 0.001**	0.542
IMA (ABSU)	0.285 (0.186-0.330)	0.444 (0.405-0.503)^§^	0.492 (0.452-0.704)^§. **. §§^	0.334 (0.166-0.426)^**. ‡‡. §§. ║║^	< 0.001	< 0.001
PON1 (U/L)	600 (215-928)	198 (131-390)^§^	316 (179-560)^‡. ║. §§^	111 (26-180)^§. **. ‡‡. §§. ║║^	**< 0.001**	**< 0.001**
Redox index	294 (202-361)	136 (98-269)^§^	89 (57-141)^§. **. §§^	73 (54-133)^§. ¶. §§^	**< 0.001**	**0.011**
Oxy score	- 0.03 (- 0.55-0.67)	- 0.06 (- 0.65-0.59)	- 0.01 (- 0.37-0.42)	0.14 (- 0.77-0.91)	0.970	0.854
Data are presented as median (interquartile range). Data were compared using the Kruskal Wallis test (P-value) + *post-hoc* Mann-Whitney U test (^†,‡,§^ P < 0.05; 0.01; 0.001 *vs.* CG; ^║, ¶, **^ P < 0.05; 0.01; 0.001 *vs.* pre-chemo; ^††, ‡‡^ P < 0.05; 0.01; 0.001 *vs.* chemo-2) and Friedman (P* value) + *post-hoc* Wilcoxon test (^§§^ P < 0.001 *vs.* pre-chemo, respectively; ^║║^ P < 0.001 *vs.* chemo-2). P < 0.05 was considered statistically significant. LTL - leukocyte telomere length. MDA - malondialdehyde. IMA - ischemia-modified albumin. PON1 - paraoxonase 1. CG - control group. SCLC - small cell lung cancer.						

**Table 3:** Telomere length and redox status parameters - survival status subgroups

	**SURVIVORS** **N = 25**				**DECEASED** **N = 35**			**S *vs*. D**	
**Parameter**	**Pre-chemo**	**Chemo-2**	**Chemo-4**	P*	**Pre-chemo**	**Chemo-2**	P**	**P^pre-chemo^**	**P^chemo-2^**
LTL	0.77 (0.59-1.05)	0.88 (0.69-1.12)	0.67 (0.57-0.84)	0.264	0.78 (0.55-0.99)	0.78 (0.61-0.98)	0.077	0.481	0.677
MDA (μmol/L)	3.556 (2.778-4.407)	3.185 (2.77-4.111)	3.185 (2.667-6.148)	0.617	0.396 (3.074-4.704)	3.926 (3.278-6.018)	0.307	0.262	0.077
IMA (ABSU)	0.431 (0.369-0.510)	0.524 (0.459-0.776)^‡^	0.340 (0.162-0.426)^†. §^	**< 0.001**	0.454 (0.415-0.500)	0.481 (0.438-0.511)	0.480	0.337	0.355
PON1 (U/L)	250 (122-543)	348 (165-588)^‡^	113 (31-182)^‡. §^	**< 0.001**	197 (129-286)	256 (195-370)	0.028	0.154	0.338
Redox index	144 (82-230)	87 (62-141)^†^	73 (52-138)^†^	**0.019**	130 (100-295)	109 (45-164)	0.136	0.495	0.871
Oxy score	- 0.09 (- 0.57-0.24)	- 0.04 (- 0.34-0.41)	- 0.06 (- 0.84-0.98)	0.986	- 0.03 (- 0.80-0.86)	0.07 (- 0.56-0.52)	0.209	0.816	0.884
Data are presented as median (interquartile range). Data were compared using the Friedman test (P*-value) + *post-hoc* Wilcoxon test (^†^*^,^* ^‡^ P < 0.001 *vs.* pre-chemo; ^§^ P < 0.001 *vs.* chemo-2); Wilcoxon test (P**-value); Mann-Whitney U test (P^pre-chemo^-value pre-chemo survivors *vs*. pre-chemo deceased; P^chemo-2^-value chemo-2 survivors *vs*. chemo-2 deceased. P < 0.05 was considered statistically significant. LTL - leukocyte telomere length. MDA - malondialdehyde. IMA - ischemia-modified albumin. PON1 - paraoxonase 1. S - survivors. D - deceased.									

All analyzed parameters, with the exception of the Oxy score, showed statistically significant differences between SCLC patients and healthy controls (P < 0.001). Malondialdehyde concentrations were significantly higher in the SCLC group before chemotherapy compared to controls (P < 0.001). Ischemia-modified albumin concentrations were highest after two cycles of chemotherapy, indicating a peak of oxidative stress at this time point. Conversely, PON1 activity was significantly lower in SCLC patients compared to healthy volunteers, with the lowest activities observed after four chemotherapy cycles (P < 0.001). The redox index, which reflects total antioxidant capacity, was also highest in the control group and gradually decreased in SCLC patients, reaching its lowest value after four chemotherapy cycles, suggesting a decline in antioxidant defense mechanisms during treatment (Table 2).

Statistically significant differences in IMA concentrations and PON1 enzyme activity were found within the survivor group of SCLC patients across different time points (P < 0.001, Table 3). Among survivors in the chemo-2 group, IMA concentrations were higher than in the pre-chemo group (P < 0.001), suggesting a possible role of oxidative stress in treatment response. In the same group of patients, PON1 activity, an important antioxidant enzyme, was also higher, particularly after two cycles of chemotherapy. In addition, the redox index was highest in the survivors in the group before chemotherapy and decreased significantly after two and four cycles of chemotherapy (P = 0.019). In deceased patients, PON1 activity varied significantly, with lower values in those who died within the first two months than in those who died after four months (P = 0.028). No statistically significant differences were observed between the survivor and deceased groups at the pre-chemo and chemo-2 study time points (Table 3).

Principal component analysis (PCA) was used to examine the relationship between a large number of parameters and the response and extensive stage. The analysis revealed three factors that explained 52% of the variance in the parameters tested (as shown in Table 4). The first factor, “Cancer Progression and Lipid Profile Factor,” primarily reflects disease severity, encompassing distant metastasis, disease stage, and HDL-C. It accounted for 20% of the variance, with positive loadings for distant metastases and stage and negative loadings for HDL-C. The second factor, “Oxidative Stress and Tumor Characteristics Factor,” captures the interplay between redox balance and tumor progression, integrating redox index, the presence of metastases, and primary tumor size. Contributing to 17% of the variance, this factor highlights a positive association with metastases and a negative relationship with redox index and tumor size. The third factor, “Gender-Specific Clinical Biomarkers Factor,” incorporates gender, LTL, and ECOG performance status. This component, explaining 15% of the variance, demonstrates positive loadings across all included parameters, underscoring their collective role in patient stratification. In addition, a univariate binary logistic regression analysis was performed to test the potential predictive power of the extracted factors for response and extensive stage (Table 5). The cancer progression and lipid profile factor was found to be a significant predictor of objective (complete/partial) response (OR = 0.278, 95% CI: 0.106-0.728, P = 0.009), progressive disease (OR = 2.305, 95% CI: 1.057-5.027, P = 0.036), and extensive stage (OR = 299, 95% CI: 2-35,100, P = 0.019), as the confidence intervals for these associations did not include 1, indicating statistical significance. For response prediction, an OR of 0.278 suggests that higher cancer progression and lipid profile factor values decrease the odds of achieving an objective response. Conversely, an OR of 2.305 for progressive disease indicates that an increase in this factor raises the likelihood of disease progression. The OR of 299 for the extensive stage suggests a strong association, but the exceptionally wide confidence interval (2-35,100) reflects substantial uncertainty, limiting the reliability of this estimate (*21*). In contrast, the oxidative stress and tumor characteristics factor and the gender-related clinical biomarker factor did not reach statistical significance, as their confidence intervals included 1, meaning they were not significant predictors of response or extensive stage.

**Table 4:** Principal component analysis extracted factors connected with objective response, progressive disease and extensive stage

**Factors**	**Variables (loadings)**	**Factor variability**
Cancer progression and lipid profile factor	Distant metastasis (0.842) Stage (0.838) HDL-C (- 0.524)	20%
Oxidative stress and tumor characteristics factor	Redox index (- 0.838) Metastasis (0.748) Primary tumor size (- 0.582)	17%
Gender-related clinical biomarker factor	Female (0.727) LTL (0.719) ECOG (0.526)	15%
HDL-C - high density lipoprotein cholesterol. LTL - leukocyte telomere length. ECOG Performance Status - Eastern Cooperative Oncology Group Performance Status.		

**Table 5:** Univariate binary logistic regression analysis for extracted predictors

**Predictors**	**Objective response**	**Progressive disease**	**Extensive stage**
Cancer progression and lipid profile factor	0.278 (0.106-0.728)^†^	2.305 (1.057-5.027)	299 (2-35100)*
Oxidative stress and tumor characteristics factor	0.940 (0.481-1.835)	1.314 (0.643-2.685)	1.587 (0.729-3.455)
Gender-related clinical biomarker factor	1.177 (0.613-2.259)	1.111 (0.587-2.104)	1.159 (0.612-2.194)
Data are presented as OR (95% CI). * P < 0.05, ^†^P < 0.01. OR - odds ratio. CI - confidence interval.			

**Figure 1:**
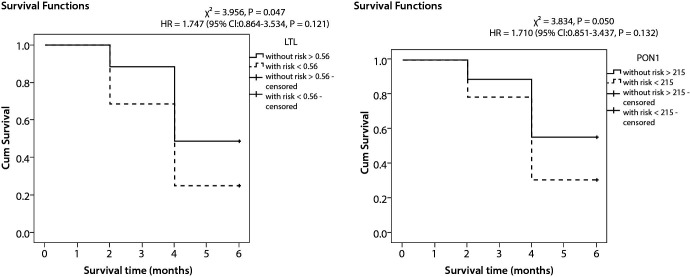
Kaplan-Meier survival analysis in SCLC patients for LTL and PON1. LTL - leukocyte telomere length. PON1 - paraoxonase 1.SCLC - small cell lung cancer.

To test the predictive power of the investigated parameters for the overall survival of patients, we performed a Kaplan-Meier survival analysis using specific cut-off values below the 25th percentile for a group of SCLC patients before starting PE therapy during the 6-month study period. Kaplan-Meier analysis revealed that lower values of LTL and PON1 may be associated with an increased risk of death (Figure 1). Kaplan-Meier survival analysis showed a statistically significant difference between LTL groups (Log-rank test: χ^2^ = 3.956, p = 0.047), suggesting that patients with LTL < 0.56 had poorer survival. However, the median survival time was 4 months in both groups, suggesting that the divergence between the survival curves is likely beyond the median point, although the distributions of overall survival are different. Therefore, when interpreting these results, it is important to consider the overall survival distribution rather than relying only on the median survival time. Similarly, the Kaplan-Meier analysis for PON1 showed a borderline significant difference in survival (Log-rank test: χ^2^ = 3.834, p = 0.050), indicating a possible trend towards poorer survival outcomes in patients with lower PON1 activity. While median survival did not differ significantly between the groups, the overall survival curves indicate a divergence, emphasizing the importance of evaluating the overall survival distribution when assessing prognosis. To further investigate these relationships, we performed a Cox regression analysis. The results suggest an increased risk of mortality for patients with lower LTL (HR = 1.747, 95% CI: 0.864-3.534, P = 0.121) and lower PON1 (HR = 1.710, 95% CI: 0.851-3.437, P = 0.132), although these results did not reach statistical significance in multivariate analysis. While these hazard ratios suggest a possible trend, the lack of significance means that additional studies with larger samples are needed to confirm the independent prognostic value of LTL and PON1 in this patient population.

The Spearman correlation analysis revealed several notable trends. A weak positive correlation was observed between LTL and PON1 activity in the pre-chemotherapy group (ρ = 0.285, P = 0.029), suggesting a possible link between telomere dynamics and antioxidant capacity during the early chemotherapy phases. In addition, the weak positive correlation between LTL and MDA in the chemo-4 group (ρ = 0.467, P = 0.022) may reflect an adaptive cellular response in which oxidative stress induces protective mechanisms such as telomerase activation or selective survival of cells with longer telomeres, rather than a direct link between lipid peroxidation and telomere elongation. Despite their weak expression, these correlations shed light on possible interactions between redox balance and telomere biology in SCLC patients.

## Discussion

Our study emphasizes the prognostic significance of LTL and PON1 activity in small-cell lung cancer. Kaplan-Meier analysis identified LTL and PON1 activity as significant predictors of survival, suggesting their potential as non-invasive biomarkers for patient stratification. While LTL provides insight into systemic telomere dynamics, its correlation with tumor tissue telomere length remains unclear. A large prospective study of 788 stage III/IV SCLC patients reported that shorter telomeres prior to diagnosis were associated with increased mortality, supporting our findings (*22*). Similarly, a meta-analysis of lung adenocarcinoma patients showed that low PON1 expression correlated with lower overall survival but better progression-free survival, underscoring its complex prognostic role (*23*).

Our results suggest that telomere attrition in SCLC is influenced by both disease progression and chemotherapy. Patients after four cycles of chemotherapy had the shortest telomeres, whereas those in the control group had the longest. Smoking, oxidative stress, and chronic inflammation are key drivers of telomere shortening, with heavy smoking history being a major risk factor in our cohort. Consistent with our study, previous reports on SCLC patients with a significant smoking history have linked shorter telomeres with poorer survival, particularly in advanced-stage SCLC (*24*). With regard to this risk factor, it is particularly important to emphasize that only one patient with SCLC in our study was a non-smoker in the past, while the remaining patients were current or former smokers.

The PE regimen exerts its effects through DNA damage and oxidative stress, possibly contributing to telomere instability. Etoposide inhibits topoisomerase II, causing DNA strand breaks, while cisplatin disrupts telomere maintenance by inducing DNA crosslinks and inhibiting telomerase activity, ultimately accelerating cellular senescence (*10*, *25*). Interestingly, we observed a transient increase in telomere length after two cycles of chemotherapy, possibly due to an initial therapeutic response and transient telomerase activation. However, after the fourth cycle, cumulative DNA damage and oxidative stress likely contributed to telomere shortening, indicating a diminishing compensatory response.

Oxidative stress plays a crucial role in the progression of SCLC and response to treatment, which is reflected in redox imbalance in our patients. Paraoxonase 1, an antioxidant enzyme associated with HDL, was significantly lower in SCLC patients than in control group, with the lowest activities observed after four cycles of chemotherapy. The PE regimen exacerbates oxidative stress, further suppressing the activity of PON1 and impairing its protective function (*26*). Consistent with our findings, Ahn *et al.* reported reduced PON1 activities in SCLC serum samples, highlighting its potential as a prognostic marker (*27*). Remarkably, our results showed the highest PON1 activity in the chemo-2 subgroup among both survivors and deceased patients, mirroring the LTL dynamics and suggesting an early adaptive response to chemotherapy-induced oxidative stress.

The increased oxidative stress in SCLC was also reflected in increased IMA concentrations and the accumulation of MDA. Ischemia-modified albumin concentrations peaked after two cycles of chemotherapy, which is consistent with the highest oxidative stress burden. These findings suggest that the chemo-2 group experienced the highest level of oxidative stress, accompanied by the strongest antioxidative response. Elevated IMA concentrations have been associated with poor survival in idiopathic pulmonary fibrosis, further supporting its potential as a prognostic indicator in pulmonary diseases, particularly those with an oxidative stress component, including SCLC (*28*). Similarly, MDA concentrations were higher in deceased SCLC patients, suggesting more severe oxidative damage. These findings are consistent with previous studies demonstrating increased MDA concentrations in lung cancer, supporting its role as a biomarker for disease progression (*29*). Furthermore, the study by Shen *et al.* suggests that cisplatin treatment in SCLC patients increases MDA concentrations, likely due to enhanced lipid peroxidation triggered by cisplatin-induced ROS accumulation, further exacerbating oxidative stress (*30*).

The redox index, a measure of oxidative balance, decreased throughout chemotherapy, with the lowest values in the chemo-4 group. This indicates a progressive weakening of antioxidant defenses during treatment. While some studies have reported an increased redox index in SCLC, our results suggest that prolonged oxidative stress weakens antioxidant capacity in SCLC patients (*31*).

Correlation analysis revealed a weak positive relationship between LTL and PON1 activity, suggesting a link between telomere integrity and antioxidant protection. Oxidative stress-induced DNA damage, inhibition of telomerase and activation of DNA damage response pathways all contribute to telomere shortening. Telomere shortening in immune cells can also impair immune function, limiting their ability to eliminate cancer cells and further worsening patients’ prognosis.

Overall, our results emphasize the intricate interplay between telomere biology, oxidative stress and chemotherapy response in SCLC. While PE treatment increases oxidative stress and contributes to DNA damage, early compensatory mechanisms, such as transient telomerase activation and PON1 upregulation, may mitigate these effects before they are overwhelmed by cumulative treatment-induced damage.

Limitations of the study include the relatively small sample size, which affected the subgroup analyzes, and the high dropout rate due to disease progression. In addition, although our control group was free of chronic diseases, some sociodemographic differences between patients and controls could not be completely eliminated. However, the focus of our study was to monitor LTL and some parameters of redox status in patients with SCLC during the study and chemotherapy cycles. Therefore, the significance of this imbalance is not considered critical to the conclusions from our study. Despite these limitations, our study provides valuable insights into telomere and redox dynamics in SCLC.

In conclusion, LTL and PON1 are proving to be promising prognostic biomarkers for SCLC survival. Their potential inclusion in non-invasive biomarker panels could improve patient stratification and enable personalized treatment approaches. Future studies with larger cohorts are needed to validate our findings and further investigate the mechanistic role of telomere biology and oxidative stress in SCLC progression and response to therapy.

## Data Availability

The data generated and analyzed in the presented study are available from the corresponding author on request.
